# Supplementary material for: Mapping the discursive dimensions of the reproducibility crisis: A mixed methods analysis
Source: PLoS One. 2021 Jul 9;16(7):e0254090. doi: 10.1371/journal.pone.0254090 (PMC8270481; doi:10.1371/journal.pone.0254090)
Supplement: S1 Table — (PDF) [file pone.0254090.s002.pdf]

S1 Table. Inter-rater reliability Kappa scores for all themes coded.

	Pairwise Kappa			
Theme	JC–NCN	KI–JC	KI–NCN	Average Kappa
2016 Nature survey	0.98	0.92	0.94	0.95
Loss of funding	1.00	0.87	0.87	0.91
John Ioannidis	0.85	0.78	0.83	0.82
Methods training	0.75	0.81	0.84	0.80
Brian Nosek/Center for Open Science	0.80	0.80	0.77	0.79
Transparency	0.82	0.73	0.75	0.77
Amgen or Bayer studies	0.80	0.68	0.80	0.76
Pre-registration	0.71	0.76	0.79	0.75
Retractions	0.76	0.78	0.60	0.71
Popular press coverage	0.66	0.67	0.76	0.70
Impact on policy or habits	0.72	0.62	0.75	0.70
Government/NGO actions	0.78	0.57	0.74	0.70
Andrew Gelman	0.90	0.63	0.56	0.70
Sample size and power	0.71	0.65	0.70	0.69
Peer review	0.63	0.60	0.82	0.68
Reagents	0.81	0.52	0.68	0.67
Economic cost	0.76	0.63	0.60	0.66
Failure to replicate important findings	0.67	0.70	0.59	0.65
General public expectations	0.69	0.70	0.56	0.65
Incentives	0.65	0.61	0.68	0.65
P values	0.67	0.61	0.64	0.64
Career costs to scientists	0.59	0.68	0.64	0.64
Heterogeneity	0.52	0.67	0.71	0.63
Bayesian statistics	0.79	0.59	0.52	0.63
Meta-science	0.79	0.54	0.55	0.63
Field differences	0.65	0.61	0.60	0.62
Fraud	0.51	0.59	0.72	0.61
Publishing culture	0.69	0.60	0.53	0.61
Legitimacy of science	0.53	0.68	0.59	0.60
excluded from analysis (average Kappa < 0.60):				
Problems with the solutions	0.59	0.61	0.57	0.59
Epistemology	0.62	0.61	0.52	0.58
Replication	0.51	0.60	0.55	0.55

Continued on next page

S1 – *Continued from previous page*

Theme	Pairwise Kappa			Average Kappa
	JC–NCN	KI–JC	KI–NCN	
Impact on medicine	0.67	0.51	0.48	0.55
Communication and collaboration	0.44	0.58	0.63	0.55
Implausible findings	0.65	0.36	0.62	0.54
Effect size	0.63	0.43	0.56	0.54
Fraud is a problem	0.41	0.53	0.65	0.53
Selective reporting	0.51	0.53	0.50	0.51
Failures to replicate	0.53	0.51	0.50	0.51
Experimental design	0.56	0.52	0.45	0.51
Personal anecdotes	0.59	0.50	0.39	0.49
Fraud is not a problem	0.30	0.46	0.65	0.47
Other statistical discussion	0.41	0.40	0.54	0.45
Other quantifying studies	0.51	0.35	0.49	0.45
Data collection and analysis	0.42	0.47	0.45	0.45
Attention in scientific venues	0.47	0.44	0.37	0.43
Bias	0.49	0.40	0.38	0.42
Evidence synthesis	0.49	0.28	0.49	0.42
Sloppy research practices	0.53	0.34	0.38	0.42
Progress of science	0.37	0.38	0.35	0.37
Regulation	0.33	0.42	0.34	0.36
Other failures to replicate	0.21	0.43	0.19	0.28
Scientists' expectations	0.30	0.30	0.23	0.28
