# Supplementary material for: Mapping the discursive dimensions of the reproducibility crisis: A mixed methods analysis
Source: PLoS One. 2021 Jul 9;16(7):e0254090. doi: 10.1371/journal.pone.0254090 (PMC8270481; doi:10.1371/journal.pone.0254090)
Supplement: S2 Table — (PDF) [file pone.0254090.s003.pdf]

S2 Table. Percentage share of the mean article profile, coordinates, contribution, and cos^2 for all themes included in the correspondence analysis.

Theme	% mean article	Dim. 1	Dim. 2	Contribution	cos^2
Transparency	9.25	-0.31	-0.09	2.12	0.06
Incentives	8.34	-0.18	-0.15	0.95	0.03
Heterogeneity	7.12	0.25	0.72	7.65	0.09
Field differences	6.28	0.31	-0.07	1.25	0.03
Brian Nosek/Center for Open Science	6.21	0.32	-0.12	1.64	0.03
P values	6.01	1.30	1.01	33.91	0.55
Publishing culture	5.82	-0.22	-0.22	1.34	0.06
Sample size and power	4.18	0.61	0.53	4.65	0.15
Fraud	4.14	-0.03	-0.51	2.46	0.06
Peer review	3.46	-0.18	-0.57	3.92	0.10
John Ioannidis	3.19	0.36	0.07	0.88	0.03
Legitimacy of science	3.15	-0.05	-0.77	4.21	0.12
Career costs to scientists	3.10	0.04	-0.42	1.36	0.03
Government/NGO actions	2.99	-0.90	0.28	6.00	0.13
Reagents	2.66	-2.66	2.18	74.06	0.84
Impact on policy or habits	2.62	-0.02	-0.92	6.65	0.14
General public expectations	2.52	0.03	-0.69	2.89	0.06
Amgen or Bayer studies	2.47	-0.56	0.22	1.82	0.05
Methods training	2.46	-0.25	-0.14	0.48	0.01
Failure to replicate important findings	2.44	0.31	-0.52	1.84	0.05
Economic cost	1.83	-0.60	0.25	1.56	0.06
Pre-registration	1.65	0.22	-0.12	0.22	0.01
Retractions	1.64	-0.11	-0.90	3.12	0.06
Bayesian statistics	1.61	2.58	2.43	30.91	0.48
Popular press coverage	1.54	0.18	-0.46	0.78	0.03
Meta-science	1.34	0.02	-0.54	0.77	0.02
2016 Nature survey	0.83	-0.22	-0.31	0.21	0.01
Andrew Gelman	0.57	1.20	0.58	2.00	0.10
Loss of funding	0.54	-0.26	-0.50	0.34	0.01
