# Supplementary material for: Mapping the discursive dimensions of the reproducibility crisis: A mixed methods analysis
Source: PLoS One. 2021 Jul 9;16(7):e0254090. doi: 10.1371/journal.pone.0254090 (PMC8270481; doi:10.1371/journal.pone.0254090)
Supplement: S1 Appendix — (PDF) [file pone.0254090.s004.pdf]

S1 Appendix: Code Book for Reproducibility Dataset

This appendix provides definitions, examples, and inclusion/exclusion rules for the thematic codes and metadata classifications applied to the articles.

Five codes were modified after all articles had been coded because low Kappa scores indicated poor inter-rater reliability on those codes.

- "Fraud is a problem" and "Fraud is not a problem" were merged into a single code, "Fraud," because of the difficulty of reliably distinguishing between passages that treated fraud as a significant contributor to irreproducibility and those that treated fraud as rare.
- "Heterogeneity/generalizability of populations/samples" and "Intrinsic complexity" were merged into a single code, "Heterogeneity," because of overlapping content between these two codes.
- "Reagents and technologies" was narrowed to "Reagents" by reviewing all passages coded with this label and un-coding those which did not mention reagents, because of the difficulty of reliably identifying what counted as a technology under this definition.

Metadata

1. **Authors**
   a) Journalist (count authors as "journalist" if that is their primary job, even if they have MDs or scientific training, e.g. Ivan Oransky).
   b) Scientist (count authors as "scientist" if they are in policy/administrative positions related to academic or industry science and have scientific backgrounds, e.g. NIH leadership).
   c) Other (e.g. member of the general public writing an opinion letter, head of a policy think tank).
2. **Date**
   a) Month/year
3. **Audience**
   a) Scientific (code as "scientific" if it is a news piece appearing in a professional society website/magazine, such as *Science, Chemical & Engineering News)*
   b) Popular (code as "popular" if the article is in a university newspaper or something for more general audiences like *STAT News* or *Scientific American)*
4. **What are the preferred terms that the article uses?**

a) If the article uses several terms in roughly equal measure (e.g. uses both "reproducibility" and "replication"), code as "mixed terms"

Stakes of the crisis

1. **Career costs to scientists**

   a) Irreproducibility will lead to scientists wasting time chasing down false leads, discourage younger generations from going into science, and damage the reputations of either the original study authors or the replicators. Particular scientists' careers will be damaged if they spend energy doing good, reproducible science while others do not.

   b) Ex. Allow scientists to publicly own up to mistakes without damage to their careers (double code with **"Incentives"**)

   c) Ex. Performing replications (that fail) of famous experiments will draw interpersonal ire from established scientists (replication bullying).

   d) *Code only for* **"Incentives"** *if it's just about the time costs of reproducible, transparent science. But code also for* ***"Career Costs"*** *if the account is drawn specifically in terms of people, like "specific scientists will get behind in their career if they spend all this time trying to document their lab practices and replicate before publishing."*

2. **Economic cost of irreproducible science**

   a) Irreproducibility wastes money, especially taxpayer money. Discussing the cost borne by pharmaceutical companies doing follow-up work on irreproducible results.

3. **Epistemology**

   a) The idea of truth itself or the scientific method is at stake, independent of its effects on policy, economics, or translational benefit.

   b) Ex. Irreproducibility is an important component of the workings of science, and is necessary to get at the ultimate truth.

   c) Ex. Irreproducibility means we are not doing proper or valid science; it hurts our understanding of what is "true."

   d) Ex. The scientific method itself is broken.

   e) Ex. We need to preserve the integrity of good science and 'depoliticize' science to produce much more objective, reliable research. (might be double coded with **"Legitimacy of Science"**)

4. **Impact on medicine**

   a) Irreproducibility may harm patients, slow progress towards developing new treatments for patients, or subject patients to substandard treatments. Include discussions of negative impact on the pharmaceutical industry under this code (double code with **"Economic cost"** if waste of pharma money is mentioned).

5. **Impact of bad science on public policy/everyday habits**

   a) Irreproducible science will lead to the creation of bad policy, or will lead people to modify their lives/everyday habits in ways that they think are supported by science but are not.

b) Ex. Reinhart and Rogoff 2010 economics article influencing politicians in UK and US for austerity policies (refuted by Thomas Herndon).

c) Ex. government actors are building climate change policy on bad, irreproducible science

d) Ex. impact of irreproducible science on legal case decisions or government regulations

e) Ex. Individuals spend time "power posing" believing that it will change their biology, even though power pose findings fail to replicate

6. **Legitimacy of science**

   a) Irreproducibility will lead to a decline in the authority and credibility of science. Include concerns that other scientists think particular subfields (e.g. psychology, economics) are not "real sciences" because of their reproducibility problems.

   b) Ex. Undermine public faith in science, as evidenced in climate change denialism

   c) Ex. credibility of particular institutions of science, like established journals and publishing venues

   d) Ex. science has become politically bent, distorted by ideological agendas permitted by bad research practices

7. **Loss of funding**

   a) Irreproducibility issues may lead to a loss of science funding from Congress, or particular research institutes.

   b) Code this only if tEx. is explicitly about potential for loss of funding, not just the implied threat from wasting taxpayer money.

8. **Progress of science**

   a) Irreproducibility will slow the pace of science, leading to a slower accumulation of knowledge or a slower process of findings translated into public benefit. Worries about science's contributions to society, interference with the pipeline from basic to applied science.

   b) Exclude discussions of hindered clinical/drug applications in the pharma industry; code that under **"Impact on medicine"** instead.

   c) Ex. the evolution of science & scientific institutions into more perfect versions of themselves

   d) Ex. the "productivity of research findings" are worsened by replication failures

9. **Stakes differ by fields**

   a) Passages that emphasize: 1) the reproducibility crisis is more severe or more problematic in some subfields than it is in others, OR 2) the stakes do not differ by fields and are common to many/all areas of science.

   b) Ex. Irreproducibility is less of a problem in psychology than medicine, because patients aren't being hurt

   c) Ex. Problems in psychology foreshadow problems in other fields; problems are not limited to psychology alone.

   d) Ex. A one-size-fits-all solution approach will not work for all scientific fields.

Signs of crisis

1. **Attention in scientific venues**

   a) Instances where articles cite high-profile individuals, organizations, or scientific journals attending to the reproducibility crisis as a sign that the crisis should be taken seriously. Or referencing attention to reproducibility in formal scientific symposia settings as a solution to the crisis.

   b) Exclude attention in the popular press, and code instead under **"Popular press coverage"**

   c) Exclude NIH/NSF action here and code under **"Government/NGO actions"**

   d) Ex. Kahneman "train wreck" email as an example of an important person speaking out/drawing attention to the issue

   e) Ex. Special issues in journals or prominent articles that address the sources/solutions of the crisis

   f) Ex. Keynote speeches about reproducibility at conferences

   g) Ex. American Statistical Association statement on p-values

2. **Failures to replicate**

   a) **Important/established findings**

      i. Failures to replicate findings that were widely assumed to be stable/credible or were textbook studies.

      ii. Ex. Failure to replicate "high profile" or "classic" studies

      iii. Ex. Replication failures of ego depletion studies, priming studies, power pose studies

   b) **Other failures to replicate**

      i. Failures to replicate findings, but not ones that were considered very important to a field or well-established. Large-scale replication projects that had high % of studies fail to replicate.

      ii. Exclude predictions that a study is irreplicable.

      iii. Ex. Dana Farber MGH oncology comparison

3. **Fraud** (*"fraud is a problem" and "fraud is not a problem" were merged to a single code for the final analysis)*

   a) **Fraud is a problem**

      i. Scientific fraud/deception/falsification is a significant issue contributing to irreproducibility.

      ii. Ex. Anil Potti fraud in cancer research

      iii. Ex. Diederik Stapel fraud in social psychology

   b) **Fraud is not a problem**

      i. Fraud is a rare problem or not a main problem contributing to the crisis

c) Double code if fraud is identified as both problematic and not that big of a deal

   i. Ex. Fraud is rare, but when it does happen, its results are disastrous

4. **Governmental/NGO actions**

   a) Actions by the National Institutes of Health (NIH)/National Science Foundation(NSF)/National Academy of Sciences (NAS)

   b) Include actions by individual NIH institutes, such as the NINDS, NCI, NIGMS, NIDA, NIAAA, NIMH, NIA. Include non-US bodies if they are similar in kind (eg. UK Academy of Medical Sciences)

   c) *This code is not mutually exclusive with **"Peer Review"** and **"Incentives"** codes*

   d) *Don't code if Collins & Tabak 2014 is cited but the sentence doesn't mention the NIH standards explicitly.*

   e) Ex. Introduction of NIH rigor and reproducibility policies

   f) Ex. NINDS conference on reproducibility in 2012

   g) Ex. Formation of NSF subcommittee on reproducibility

   h) Ex. government agency funding for open source data sharing/coding platforms

5. **Implausible findings**

   a) An implausible finding produced using established methods alerts people that there must be something wrong with those established methods.

   b) Ex. Bem's extrasensory perception study

   c) Ex. Chronological rejuvenation to a Beetle's song

   d) Ex. Dead fish fMRI study

6. **Personal/individual anecdotes**

   a) A personal story of what happened to the individual speaker/author, such as a failure to replicate a finding within their own lab, where the personal and emotional experience is at the core of the example—an "it happened to me" story. Also code under this if the author writes about another scientist's failed replications in an anecdotal, narrative way. Often occurs in quotes and personal blog posts.

   b) Ex. "I" narratives about arduous attempts to replicate other findings and having lots of trouble contacting original authors, getting access to data.

7. **Popular press coverage**

   a) Narratives pointing to articles or books in the popular press as a sign that there is a reproducibility crisis.

   b) Ex. "Trouble at the Lab," *The Economist* article, 2013

   c) Ex. "A Sharp Rise in Retractions Prompts Calls for Reform," *New York Times*, 2012

   d) Ex. "The Truth Wears Off," *The New Yorker*, 2010

8. **Quantifying problems**

   a) **2016 Nature survey**

i. Monya Baker's "1,500 scientists lift the lid on reproducibility" article

ii. *Code under this even if the study is not described in the tEx. of the article, but the article makes an assertion about the extent of the crisis and backs it up with a cite or link to this survey.*

b) **Other studies quantifying problems**

i. Studies that try to put a number on the extent of a particular problem that contributes to irreproducibility. Code even if the article does not mention specific paper, but references generic 'scientific research' providing some quantified number related to reproducibility.

ii. Exclude Ioannidis 2005 study and code under **"Ioannidis"** instead

iii. Exclude Amgen/Bayer studies and code under **"Amgen/Bayer studies"** instead

iv. Exclude Nosek papers and code under **"Nosek"** instead

v. Exclude *Nature* 2016 survey and code under **"Nature 2016 survey"** instead

vi. Ex. Turner 2008 on selective publication and estimates of drug efficacy

vii. Ex. Kilkenny et al 2009 on quality of animal studies

viii. Ex. Fanelli 2010, 2011 on increase in positive results

ix. Ex. Using meta-analysis as technique to show extent or source of reproducibility/methodological problems related to reproducibility

c) **Amgen or Bayer studies**

i. Reference to Prinz et al. 2011 "Believe It or Not: How Much Can We Rely on Published Data on Potential Drug Targets?", and Begley and Ellis 2012 "Raise standards for preclinical cancer research"

9. **Reformers**

a) **Andrew Gelman**

i. Code where Gelman or Gelman's work is mentioned as an indication that there is a crisis, or where Gelman is cited as a reformer/qualified commentator on the crisis.

b) **Brian Nosek/Center for Open Science**

i. Code whenever Nosek's work is cited. Include mentions of The Reproducibility Project: Psychology and The Reproducibility Project: Cancer Biology; the 2015 Open Science Collaboration paper about the psychology project or coverage of that paper; and the Many Labs projects.

ii. Use this code exclusively when coding Nosek/COS stuff, i.e. don't double code with **"Replication"** or **"Transparency of Data"** (unless they are included in a list of separate things).

iii. *Note that the Social Sciences Replication Project and Science Exchange are not part of the Center for Open Science.*

c) **John Ioannidis**

i. Code whenever Ioannidis or his work (even if not explicitly reproducibility-related work) is quoted/referenced. Code even if the article doesn't mention him by name, but cites/quotes his work (eg. 2005 "Why most published findings are false" article) as a sign that there is a reproducibility crisis.

ii. *Double code with **"Meta-science"** if pertinent, like when the article discusses Ioannidis's work in a larger field that studies science itself.*

10. **Retractions**

a) Increasing number of retractions from journals, slowness to retract things from the literature, and lack of visibility of retractions are signs of a problem. Include passages about the need for better, more consistent oversight measures to retract faulty articles.

b) Ex. organizations dedicated to retraction like Retraction Watch are trying to combat the invisibility of retractions

c) Ex. Several of Wansink's major papers had to be retracted due to misreporting

d) *Double code with **"Fraud"** if they are mentioned together*

Sources of trouble/solutions

1. **Change expectations about science**

a) **General public's expectations**

i. The general public needs to realize that studies are not the "absolute truth," and that irreproducibility of a study does not imply incorrectness of theory. Also code for passages about how non-scientist professionals, like judges, need to be more skeptical of findings from academic labs.

ii. *If it is unclear who the changing expectations message is directed at, differentiate by source (i.e. code under **"General public's expectations"** if it is an article aimed at the general public, and under **"Scientists' Expectations"** if it is an academic article)*

iii. Ex. General public needs to be more aware of the reproducibility crisis and the limitations of modern science

iv. Ex. General public needs to be better educated in their understanding of how science really works (target K-12 schools) (including their statistics education)

v. Ex. Science journalism has an important role to play in making sure that the public gets these messages, and ensuring that journalism does not distort or sensationalize science. Scientists themselves need to be wary of sensationalizing the claims they make about scientific research/studies to the general public.

b) **Scientists' expectations**

i. Change 1) expectations about what degree of reproducibility or what type of reproducibility is to be expected, 2) unreasonable expectations about the reliability of small studies, 3) expectations about how noisy data is hard to reconcile with theories, 4) expectations about the pace of scientific discoveries. Include assertions that scientific funders or pharma need to change their expectations, not just scientists.

ii. *If it's about journals/funding agencies changing their 'novelty' standards related to the incentive structure that's driving scientists, code only under* ***"Incentives."***

iii. Ex. Change expectations about what a failed replication means—it does not necessarily mean a 'falsification' of the unreplicated theory or a mark of failure. Instead, it can mean the replicating lab made a mistake or the original effect is a true effect that only works under specific conditions. Or it can mean an 'opportunity' to expand knowledge in some other way (a replication failure is a good thing).

iv. Ex. Dead ends should be expected, highly novel results should be considered less likely to be reproducible.

v. Ex. Science is not self-correcting just by virtue of internal exchange among scientists. Robust self-correction mechanisms do not exist like people suggest.

vi. Ex. Scientists have too high or uncritical expectations of "black box" statistical tools (double code with **"Other Statistical Discussion"**).

2. **Communication and collaboration**

   a) Developing new methods for promoting communication and collaboration between different groups.

   b) *Exclude exchanges of materials and code under* ***"Reagents/technologies"*** *instead*

   c) Ex. Multi-sited studies.

   d) Ex. Deeper collaboration between pharma and academia.

   e) Ex. Issues with communicating and teaching protocols between original lab and replicating lab.

   f) Ex. improving the specificity of common terms like 'replication' to ensure scientists are talking about the same thing.

   g) Ex. Funding agencies need to collaborate to develop common policies and requirements for applicants, who likely seek funding from multiple sources.

3. **Evidence synthesis**

   a) Lack of systematic review or integration of existing evidence, calls for "weight of evidence" evaluations or meta-analysis. Critiques of using one type of methodology and calls for using multiple sources or methods to triangulate evidence.

   b) Ex. The creation of scientific theory from multiple types of evidence (conflicting or not).

   c) Ex. Scientific theory involves the integration of a vast array of results, including conflicting or irreproducible results, before being able to make a generalized account.

4. **Heterogeneity/generalizability of populations/samples** *(this code was combined with "intrinsic complexity" in the final analysis)*

a) Overly standardized or narrow populations/conditions/models lead to poor replication/generalizability/external validity, should use more multi-lab replications (i.e. solving homogeneity with heterogeneity). Alternatively, populations or protocols that are too variable (e.g. diverse cage & lab environments for model organisms) need to be further standardized to get more precise results (i.e. solving heterogeneity with homogeneity). Sentiments that small factors affecting experimental measurements need to be recorded, standardized, attended to in order to account for variability.

b) This code is for tEx. that frames problematic factors of heterogeneity/homogeneity as being solvable.

c) Ex. systematic herogenization of lab environments

d) Ex. Cross-cultural research–either to make more generalizable claims or to be aware of the specificity of your claims

e) Ex. Inclusion of children/elderly/women in research

f) Ex. animal models that only replicate one feature of a disease and don't provide good predictions for how a drug will behave in human populations

5. **Incentives**

a) Incentives in the career pathways of scientists (or in science/industry broadly speaking) are encouraging problematic behavior; incentives need to be realigned to correct reproducibility problems. Distorting incentives might include pressure to publish, to get grants, to get tenure, excitement of/desire for novel results.

b) *Include discussions of differing incentives between science and industry, and double code with **"Stakes Differ by Fields."***

c) *Exclude when it's talking about incentivizing replications, and code under **"Replication"***

d) Ex. Change criteria for what a high-quality researcher is

   i. Ex. PQRST evaluation metric for researchers

   ii. Ex. reproducibility index

e) Ex. Change tenure incentives, not emphasizing publication in high-impact journals or the number of studies published

f) Ex. Changing incentive structure within grant mechanisms (such as longer timeline) to resolve existing funding pressures

g) Ex. Decreased funding opportunities for scientists throws off "good" competition, making the environment much more hostile and competitive, therefore incentivizing questionable research practices

6. **Intrinsic complexity** *(this code was combined with "Heterogeneity/generalizability of populations/samples" in the final analysis)*

a) Irreproducible data arises because of the intrinsic complexity of the phenomenon under study (rather than the cause being sampling problems, design of animal models that limits generalizability, or some flaw in the research design or analysis). This intrinsic complexity is often a type of "unknown unknown." This code is for situations in which the article implies that the complexity cannot be resolved or regulated.

b) Ex. Sometimes effects just fade away over time because of ‘cosmic habituation’

c) Ex. Complexity of gene–environment interactions

d) Ex. Individual variation in model organisms in emotional states and stress responses affect behavioral/phenotypical measurements

e) Ex. Small differences in experimental context, like the testing room, and experimenter’s mood can have big impacts on the effect size

f) Ex. Tiny differences in experimental technique, like rocking v. shaking or the average temperature of lab, affect replication success & effect size

7. **Journals and publishing culture**

   a) How journals, editors, and overall publishing culture contribute to irreproducibility problems. How journals can function as gatekeepers or enforcers of standards.

   b) *Sometimes double code with **“Nosek/COS”** if the article calls for changes in journal/publishing culture and cites Nosek or COS*

   c) Ex. *Basic and Applied Social Psychology* banning the use of p-values

   d) Ex. *Nature* checklist of reporting standards

   e) Ex. Allow publication only after an exploratory study and confirmatory study have been done.

8. Ex. Journals are strained enough and don’t have the resources/bandwidth to critically parse through the messiness of data that are not communicated in ‘pretty’ narratives.

   a) Ex. Journal impact factor is criteria for success, which allows for “gaming” behavior to increase impact factor (double code with **“Incentives”**).

9. **Meta-science**

   a) Code even if the article doesn’t use the specific term “meta-science” but describes something like a discipline-independent field (or group of researchers, or institute) devoted to thinking about scientific practice. Include any reference to “metaresearchers” or “metaresearch” or “meta-experts.”

   b) Ex. Empirical research on the effectiveness of particular policy changes and interventions within science

   c) Ex. “Rigorologist” hires

   d) Ex. (METRICS) Meta-Research Innovation Center at Stanford

   e) Ex. Research-integrity advisors hired by research institutions to evaluate the integrity and quality of researchers’ works

10. **Peer review**

   a) Anything that has to do with having other experts reviewing publications, research plans, or grants; flaws with the current system and how it should be improved; funding agencies evaluating proposals. Include suggestions for new or informal methods of peer review.

   b) *Exclude if it is about potential revenge in the grant review process and code as **“Career costs to scientists.”***

c) Ex. More detailed and strict peer review in journals

d) Ex. Peer prediction market

e) Ex. Peer reviewers should be paid experts.

f) Ex. Pre-study peer review, such as Registered Reports through Center for Open Science

11. **Problems with the solutions**

a) Complaints that proposed solutions to enhance reproducibility are infeasible, not cost effective, or will not be enforceable/well-followed. Complaints that the way that solutions have been implemented are lacking or too one-size-fits-all. Often takes the general form: "X solution is good, but insufficient to solve Y problem because of reasons Z1, Z2, etc."

b) *Double code with other problems/solutions codes, where it seems appropriate*

c) *Exclude meta-science initiatives that may uncover problems with the solutions, but are not general complaints about the solutions (code instead under **"Meta-science"**).*

d) Ex. Descriptions of systematic efforts to replicate and how they've failed (such as the Amgen paper not revealing the titles of irreproducible papers—note that this would also be double coded with **"Transparency"**)

e) Ex. Replications do not address the deeper root of the problem, the fact that psychology does not have well-developed theories

f) Ex. Even if we get scientists to do more replications, the field has not figured out how to interpret what the results of replications mean.

g) Ex. Even when a study is found to be irreplicable, it stays in the literature for a long time and is difficult to remove.

12. **Reagents or technologies** *(this code was modified in the final analysis to include only references to reagents, and not other kinds of technologies)*

a) Problems with particular reagents (eg. cell lines, antibodies), technologies (eg. genotyping platforms, microarrays), or drug assays and protocols. Ways to fix these problems. This code refers to known problems with standard technologies and tools being used in experiments (tools that are taken for granted which need to be validated to ensure they're working as intended).

b) Exclude [software glitches, bad measurements in psychological tests (poor indicators, no standard definitions of behaviors), problems with behavioral phenotype tests, and variability in mouse lines.]

c) *Double code with **"Transparency"** if it is about needing detailed reporting of technology versions and specific reagents used.*

d) Ex. efforts to use the exact same reagents and materials (in replication experiments)

e) Ex. check antibodies and cell lines for contamination

13. **Regulation**

a) New or stronger requirements and regulations for reproducibility standards, enforced by an institution of some kind, not cultural norms/informal/voluntary changes.

b) *Include under this code if the solution proposes that the NIH or another institution should do something, but it's theoretical.*

c) *Exclude publishing requirements and code under* ***"Journal/publishing culture."***

d) *Exclude actual actions that governments/NGOs has already taken or is very likely to take, and code under* ***"Governmental/NGO actions"*** *instead*

e) Ex. Research institutions should mandate open data access

f) Ex. increased oversight for potential misconduct, led by IRBs or independent investigative unit

g) Ex. Lab notebook auditing

h) Ex. Problems with ethical review board regulations that encourage smaller sample sizes for model organisms, which contribute to low power and low reproducibility.

14. **Replication**

a) Comments about the lack of replication in current scientific practice, the need to incentivize more of it, ways to fund replications, and examples of successful large-scale replication endeavors or platforms.

b) *Exclude Center for Open Science stuff, and code that instead under* ***"Nosek/COS"***

c) Ex. Labs should replicate their own work before attempting to publish it.

d) Ex. Science Exchange service for performing replications.

e) Ex. Create journals specifically for publishing replications (double code with **"Journals/publishing culture"**)

f) Ex. Need clear standards for what constitutes an appropriate replication, different kinds of replications.

g) Ex. Conceptual replications are insufficient to determine which research is irreplicable, and are similarly unpublishable. Not enough incentives for direct replications.

15. **Reporting**

a) **Selective reporting**

i. Lack of publication of null results; bias towards publication of positive results; the "file drawer" problem; general suggestions that fields need to become more receptive to null/negative results. Code passages that discuss general publication bias, and do not code under **"Bias"** code.

ii. Ex. need to create venues where null results can be published (double code with **"Journal/publishing culture"** if the role of journal editors is emphasized)

iii. Ex. PsychFileDrawer

iv. Ex. Journals/scientists favor telling the "perfect story," artificially tidied results, and inflated import. (double code with **"General public's expectations"** if it's about scientists trying to give inflated versions of their results, giving the public misconceptions of science.)

b) **Transparency of data and methodology**

i. Calls for "open science" or ways to make data and methodology more accessible to people other than the original creators; calls for more transparent and complete reporting of different aspects of methodology in publications. Include problems with transparency of funding sources and poor documentation/lab notebook practices.

ii. *Double code with **"Problems with Solutions"** if systematic efforts to replicate experiments were not transparent enough about their methodology, data sets, etc.*

iii. Ex. the need to create new reporting standards or enforce existing ones (eg CONSORT, ARRIVE, EQUATOR standards) (double code with **"Journals & Publishing Culture"** if journals are the ones enforcing ARRIVE standards or something similar)

iv. Ex. Make raw data publicly available

v. Ex. Use digital notebooks to track data, methods, people (good data record keeping, like version control)

vi. Ex. Journals should allow for more extensive descriptions of methods (double code with "**Journals/publishing culture**")

16. **Research methods**

a) **Bias**

i. Ways in which various forms of cognitive bias (e.g. confirmation bias, anchoring bias, selection bias, groupthink) or general human bias can contribute to irreproducibility. This code's purpose is to capture forms of bias that other, more specific codes do not already encompass. For example, issues with experimental blinding should go under **"Experimental Design"** code, and not under this code.

ii. Ex. A PI's bias toward his/her "pet theory" that they have spent a long career developing and refining

iii. Ex. conflicts of interest from funding sources, personal connections that could distort results

iv. Ex. groupthink in the scientific establishment

b) **Data collection and analysis**

i. Ways in which data collection and analysis practices can contribute to irreproducibility.

ii. Exclude analysis problems that are about statistical issues

iii. Include questionable research practices (QRPs) *except* 1) if pejorative in tone, in which case code under **"Sloppy Research,"** or 2) if it's about p-hacking or strongly implying the idea of p-hacking, code only under **"P-values"**

iv. Ex. Need high integrity, complete data collection processes, especially when working with big data processes or lots of data collected over time (like when testing iterations of drugs)

v. Ex. Continuing to collect data points to find a significant result, or stopping collection once a significant result is obtained

vi. Ex. "Researcher degrees of freedom"

vii. Ex. HARKing (Hypothesizing After Results are Known)

viii. Ex. blind data analysis (writing all analyses on a machine-generated, scrambled data set and then applying all those analyses to the real data set)

c) **Experimental design**

i. Ways in which poor experimental design and structure can contribute to irreproducibility; potential solutions to improve experimental design.

ii. Ex. Lack of proper randomization and controls

iii. Ex. More blinding in studies

iv. Ex. Computer-based tools for experimental design

v. Ex. researcher's choice of operationalization of variables (e.g. how the researcher actually measures something abstract like health outcomes in a hospital)

vi. Ex. strong inference tests, explicitly developing a competing hypothesis and designing experiments to differentiate between the two

d) **Pre-registration of study protocols**

i. Calls for pre-registration of papers as a solution.

ii. Ex. Center for Open Science Registered Reports

iii. Ex. Locked methodology and statistical tests

e) **Sloppy research practices**

i. Statements that have a pejorative tone about how researchers are "sloppy," "careless," or using "poor" methodology; statements implying that scientists simply need to adhere to scientific common sense rather than doing something radically different or new.

ii. Ex. "It is astonishing that scientists do not do this or that in their experimental practices. . . "

iii. Ex. culture of 'statistical rituals' in which scientists do not use common sense about basic statistics

iv. Ex. Highly pejorative discussions of questionable research practices

f) **Training in research methods**

i. Lack of research methods training, especially for junior scientists, and need for new training opportunities. Include training in formal and informal education settings.

ii. *Include training in statistics and double code with* ***"Statistical methods"*** *only if the passage discusses specific changes to statistical practice.*

iii. Ex. PIs should mentor junior scientists more closely, provide greater oversight of the experiments done in their lab

iv. Ex. Training in prudent scientific practice, like validating scientific reagents (double code with **"Reagents"**)

v. Ex. Labs with sloppy research methods get ahead and pass on their practices with their lab offspring

vi. Ex. Textbooks used in coursework do not accurately represent the controversies surrounding certain major findings.

17. **Statistical methods**

a) **Bayesian stats**

i. Over-reliance on frequentist statistics as a source of irreproducibility, suggestions that Bayesian approaches can help solve the problem.

ii. Ex. use of Bayesian priors

b) **Effect size**

i. Problems with declining effect sizes over time in the literature; artificially inflated effect sizes in the literature due to publication bias (double code with **"Selective reporting"**).

ii. Ex. Reduction in efficacy of treatment after consideration of unpublished trials

iii. Ex. calculated replicability of experiments should not depend on the effect size

iv. Ex. Because of the history of landmark advancements, scientists now seek smaller and smaller effects in their experiments, contributing to the difficulty of picking out subtle effect sizes from all the noise

c) **Other statistical discussion**

i. Discussions of statistical problems/solutions that do not fit well into other statistics code categories.

ii. Ex. Scientists should report confidence intervals

iii. Ex. Automatic, mechanical execution of statistical tests without exercising appropriate judgment

iv. Ex. Attention to statistical rigor needs to be incorporated into everyday culture

v. Ex. Need for better statistical tools & packages to allow scientists with basic data science training to do robust, reproducible stat analysis

vi. Ex. a priori inferences about sample/population means and standard deviations

d) **P-value problems and changes**

i. Discussion of problems with the use of a 0.05 alpha level and interpretation of p-values by scientists or lay public; discussions of p-hacking and false-positive manipulation; proposals to stop using p-values and null hypothesis significance testing as a method. Code if popular articles talk about p-value issues in plain language without mentioning the term specifically (such as "significance" or "null hypothesis" testing or "false-positive" data manipulation).

ii. *Double code with **"Data collection/analysis"** if pertaining to the ways in which Researcher Degrees of Freedom can inflate type I errors/false-positives that seem in line with p-hacking.*

iii. Ex. Different p-value standards for different fields

iv. Ex. Need explicit declaration of "No p-hacking" in each paper

v. Ex. Multiplicity issues leading to high false discovery rate

vi. Ex. data-dredging, data-fishing for a low p-value

e) **Sample size and power**

i. Claims that most studies use sample sizes that are too small and are therefore under-powered; calls to increase sample sizes.

ii. Ex. Compute necessary sample size beforehand and justify the quantity
