# Supplementary material for: Mapping the discursive dimensions of the reproducibility crisis: A mixed methods analysis
Source: PLoS One. 2021 Jul 9;16(7):e0254090. doi: 10.1371/journal.pone.0254090 (PMC8270481; doi:10.1371/journal.pone.0254090)
Supplement: Attachment — Submitted filename: PLOS ONE response to reviewers.docx [file pone.0254090.s005.docx]

**Editor:**

1. **Complete COREQ or equivalent reporting checklist.** A completed SRQR checklist has been submitted with the revised manuscript. The COREQ checklist is formatted for interview-based qualitative studies, and so we opted to use the SRQR checklist instead.
2. **Address formatting issues.** We have re-generated the bibliography in PLOS ONE format.
3. **Explain why some data is not publicly available.** We have made the data and code needed to reproduce the analyses in the manuscript available on GitHub. This includes the outputs of the qualitative data analysis performed using NVivo, in spreadsheet format. We have not included the original NVivo file since this file contains the full text pdfs of the articles we analyzed and making this publicly available may constitute a copyright violation.
4. **Clarify the status of the *Review of General Psycholog*y manuscript.** The related manuscript has been accepted with minor revisions at RGP but has not yet been published. We have added a citation to this forthcoming paper in the revised text. As stated previously in the submission letter, the RGP manuscript is based on the same data set but analyses this data using a different technique (subset correspondence analysis), answers a different research question (how have reproducibility conversations changed over time?), and is intended for a different audience (historians and philosophers of psychology). It does not constitute dual publication because very little text is shared between the two articles, apart from the methodological description of the sample collection and coding process.

**Reviewer One:**

1. **The initial assumption that reproducibility conversations are ill-structured is not supported by references to existing research.** We have added multiple references to the introduction to better support this claim.
2. **It is difficult for readers unfamiliar with CA to “benchmark” the results**. The point is well-taken that the correspondence analysis is less meaningful without some background expectation, or what the CA would look like under greater heterogeneity. It is difficult to provide quantitative benchmarks since the numeric scales produced through CA cannot be meaningfully compared across data sets, but we have added additional text at the beginning of the results section that describes qualitatively what our results may have looked like if reproducibility discourse was more heterogeneous.
3. **The rationale for focusing on biomedicine and psychology is not clear.** We have added additional text to the Materials and Methods/Data collection section to explain that we selected biomedicine and psychology because reproducibility issues in these fields have been discussed extensively both in the scientific community and the popular press.
4. **The findings may suggest that reproducibility research might constitute a distinct discipline of its own.** This is an interesting suggestion, which we now take up in the revised conclusion. We now argue that while the presence of a shared discourse may be partially explained by the emergence of metascience as a discipline, but not fully, since the shared discourse extends beyond the community of authors who self-identify as metascientists.
5. **Include quotations and examples from the texts analyzed**. We appreciate this invitation to expand the presentation of our results in this direction and have included more representative quotes throughout the results section.
6. **Provide a clearer explanation of how the sampling strategy employed is useful for addressing the research question posed.** We appreciate the feedback on our description of the sampling strategy. We have added additional text describing why purposive sampling strategies are common in qualitative research, and why maximum variation sampling is especially appropriate for characterizing the range and variability of reproducibility narratives.

**Reviewer Two:**

1. **The manuscript conducts statistical analyses of variables derived from word searches of an arbitrarily selected body of texts.** We regret that we failed to convey to this reviewer key pieces of our methodological approach. First, the frequencies are not based on word searches, but on a qualitative coding scheme that we have now described in greater detail, both in the Methods section and in the S1 Appendix. Second, the texts are not arbitrarily selected, but have been selected through maximum variation sampling. We have expanded our description of this sampling strategy, as described above in R1.6.
2. **The analysis “statistical/computational exercises on their own applied to methodological/philosophical discussions of the nature of science... can do almost nothing to clarify what are inherently abstract disputes over the nature of science.”** We cannot answer the question of whether computational techniques alone (e.g., topic modeling/natural language processing) can be useful in clarifying reproducibility discussions, since this is not the approach that we employed here. Our mixed methods approach uses qualitative techniques that are appropriate for parsing the different positions taken by actors within a social world, in combination with quantitative approaches to visualize the results of that analysis. We suspect that this concern is rooted in the misunderstandings identified in R2.1 above.
3. **The decision to normalize the counts of coded text using the total amount of coded text (rather than using raw counts or normalizing the counts against the total length of the text) is inappropriate.** We have added additional text to explain why this choice is appropriate. As explained in the Abdi and Williams (2010) article that we cite, using PCA in our case would fail to reveal meaningful similarities in style between authors because of the differences in the length and format of the texts.
4. **The findings presented in the manuscript could be derived through alternative methods such as systematic review, and the use of correspondence analysis is “lacking in any real substance or intrinsic motivation.”** We agree that it may be possible to arrive at similar results using alternative methods, but it does not follow that there is no value to the methods we use here. Given the large number of texts, themes, and metadata variables we analyzed, exploratory data analysis techniques are helpful for summarizing and visualizing the main features of the data set. We believe that the results of our analysis are more succinctly presented through visualizations in combination with narrative than they would be through a narrative format alone.

**Reviewer Three:**

1. **The manuscript needs a more thorough description of the article selection method, including rationales for the selection of databases, selection of search strings, and how maximizing variation along some dimensions (e.g. year of publication) may reduce variation in others (e.g. the appearance of multiple articles by the same authors in the data set).** We have added more detail on the rationales for selecting the fields studies, databases employed, and search strings used to the methods section (see also R1.3). We have also added a note to the methods section acknowledging that maximizing variation in some areas may have reduced variation in others.
2. **The number of articles is smaller than expected given the scale and scope of reproducibility discussions**. We have added a citation to Fanelli (2018), which describes a bibliographic data set generated using search terms that are comparable to ours, and which identifies only 99 publications related to the reproducibility crisis.
3. **The process for deriving the thematic codes is not clear and involves many “degrees of freedom.” Some expected codes (e.g., “harking”/”forking,” “open science”) do not appear on the list**. We have added additional text to the methods section to describe the process of code development through grounded theory methodology, including the important distinction between the “open coding” phase where codes are generated, and the “focused coding” phase where established codes are applied to the body of texts as a whole. We hope that this alleviates some of the concerns about researcher degrees of freedom, although we also note in the revised methods text that some of these concerns may arise from differences inherent to quantitative and qualitative research paradigms. We have also incorporated the code book (originally provided as a standalone document on the GitHub repository) as a new appendix to the paper (S1 Appendix). The code book describes in detail what was included under each code, which will help readers identify how themes of interest were parsed according to our scheme (e.g., discussions of open science would be classified under “transparency of data and methods” in our analysis).
4. **The rationale for performing the text search queries is not clear.** We have added additional text to the Materials and Methods section to clarify the purpose of creating the word frequency variables, which serve as a check on the qualitative analysis and to enhance the interpretation of the CA dimensions.
5. **The Kappa values of many thematic codes appears unexpectedly low and raises concerns about the exclusion of these codes from the analysis**. Low Kappa scores are very common in qualitative research, particularly as codes increase in conceptual complexity and for codes that appear at low frequency in the corpus. We have added additional text in the Materials and Methods/Qualitative data analysis section to clarify why it is difficult to achieve strong Kappa scores. We have also added text to the discussion section to explain that the CA mapping presented here should not be interpreted as a definitive mapping, since the configuring of the map would shift depending on the codes included (or if a different coding scheme altogether were used).
6. **The methodology was not pre-registered.** We agree that this is a limitation. At the time we began this study, we were not aware of any options for pre-registration that were tailored to qualitative research. The OSF, for example, released its first template for pre-registration of qualitative research in September 2018, very shortly after we began our study. To compensate for this limitation, we have attempted to be as transparent as possible in reporting our methods and data.
7. **The rationale for the expanded interpretation of Dimensions 1 and 2 is not clear.** We are grateful for the encouragement to revisit our interpretations here because in rechecking our analysis we discovered an error in the r value originally reported for the “psychology” word frequency variable, which suggests that our original interpretation that Dimension 1 was also capturing differences in discipline was not as sound as we had initially thought. Accordingly, we have revised the manuscript so that Dim 1 is presented as separating bench vs. statistical techniques and Dim 2 is interpreted as separating social vs. technical issues. We still discuss the relationship of these two dimensions to discipline and audience, but are now more careful in our presentation of the potential weaknesses of these interpretations.
8. **The dimensions have not been labeled in Figures 5 and 6**. **A discussion of Dimension 1 in Figure 6 should be included for completeness**. We have corrected these omissions.
9. **A robustness/bootstrap analysis would provide insight into how the sampling method has impacted the results**. We thank the reviewer for this generative suggestion. We have implemented a bootstrap analysis by using the same multiple factor analysis design used to analyze articles by author and by audience. This analysis does demonstrate the impact of the sampling strategy on the results, but it has also allowed us to identify additional clusters of themes at the core of the conversation that can be differentiated with confidence. The results of this new analysis are presented in Fig 5 of the revised text.
10. **It is unclear whether the results of the analysis can be generalized to the population as a whole**. We have added additional description about the sampling strategy (see also R1.6) as well as a general caveat to the beginning of the methods sections about what kinds of conclusions can be generalized to the population as a whole, and what kinds of conclusions cannot be drawn from our analysis. We have also added several caveats to the discussion section to remind readers of these limitations.
11. **The manuscript could explore how the discursive dimensions might have changed over time**. We address this question in a separate manuscript: the forthcoming *Review of General Psychology* manuscript described in point Editor.4 above.
